# Wide Diurnal Temperature Amplitude and High Population Density Can Positively Affect the Life History of *Sitobion avenae* (Hemiptera: Aphididae)

**DOI:** 10.1093/jisesa/ieab011

**Published:** 2021-03-10

**Authors:** Kun Xing, Dongbao Sun, Jianzhen Zhang, Fei Zhao

**Affiliations:** 1 Shanxi Key Laboratory of Integrated Pest Management in Agriculture, College of Plant Protection, Shanxi Agricultural University, Taiyuan, Chin; 2 Shanxi Shouyang Dryland Agroecosystem, National Observation and Research Station, Shanxi, China; 3 Institute of Applied Biology and College of Life Science, Shanxi University, Taiyuan, China; 4 Institute of Environment and Sustainable Development in Agriculture, Chinese Academy of Agricultural Sciences, Beijing, China

**Keywords:** population density, thermal amplitude, life history, English grain aphid, synergistic interaction

## Abstract

Diurnal temperature amplitude is known to have a large influence on insect life history. Population density affects intraspecific competition and many other aspects of insect life history. However, there is limited information on the interactive effects of these factors on insects. Here, we tested the interactive effects of three diurnal temperature amplitudes (22 ± 0°C, 22 ± 6°C, and 22 ± 12°C) and three population densities on the development, survival, longevity, and fecundity of the English grain aphid *Sitobion avenae* (Fabricius) (Homoptera: Aphididae). At a constant temperature, increasing population density reduced the growth and survival of early-instar nymphs, increased longevity, and reduced fecundity. At a low population density, increasing temperature amplitude inhibited nymph development. However, even at a high temperature amplitude, nymph survival rate was higher than expected, and reproduction was possible because the recovery of the lower night-temperatures eliminated thermal stress. Increasing the population density reduced, and even reversed, the negative effects of the wide temperature amplitude. This may reflect synergistic interactions between population density and wide temperature amplitude as these stressors each incur energetic costs. These findings emphasize the importance of temperature amplitude and population density for improving prediction accuracy and damage assessment during pest control modeling.

Insects often experience diurnal temperature variability (Lambrechts *et al.* 2011), and it is important to assess how affects their life history (Huey and Kingsolver 1989, Vázquez et al. 2017). Constant temperature experiments cannot reflect the impacts of natural diurnal temperature variability on insects because they do not account for the direct effects of daily maximum and minimum temperatures or acclimation effects induced by temperature variability. Constant temperatures and variable diurnal temperatures have quite different effects on development (Niederegger *et al.* 2010, Verheyen and Stoks 2019), reproduction (Terblanche *et al.* 2010, Carrington *et al.* 2013), survival (Colinet *et al.* 2011, Cavieres *et al.* 2018), thermal tolerance (Terblanche *et al.* 2010, Salachan and Sørensen 2017), behavior (Paaijmans *et al.* 2010, Miyazaki *et al.* 2011), and even gene expression (Wang *et al.* 2006, Sørensen *et al.* 2016) in insects. Further, in temperature-shock and heat-hardening experiments, the high and low temperatures used are close to the maximum and minimum daily temperatures, respectively. In these studies, insects are exposed directly to the high or low temperatures, immediately after experiencing their rearing temperature (Yoder *et al.* 2009, Roux *et al.* 2010, Xing *et al.* 2015b). However, temperatures under field conditions change slowly and gradually, not rapidly. Further, thermal tolerance varies with the rate of temperature change (Mitchell and Hoffmann 2010). Therefore, constant temperature and temperature-shock or heat-hardening methods are not appropriate for elucidating how temperature variability affects insects.

Population density regulates the strength of intraspecific competition and affects many aspects of life history, as well as morphological and physiological characteristics of a species (Niemelä *et al.* 2012, Fitzgerald *et al.* 2013, Schou *et al.* 2013, Onufrieva *et al.* 2019). The development rate is increased by high population density (Applebaum and Heifetz 1999), such as *Diploptera punctate* (Woodhead and Paulson 1983), *Mamestra brassicae* (Goulson and Cory 1995), *Diabrotica virgifera* (Barnes and Siva-Jothy 2000), and *Chironomus riparius* (Hooper *et al.* 2003). High population density is also known to increase wing size (Cease *et al.* 2010), body length, and the development of the mesothorax (Morooka *et al.* 1988, Fujisaki 1989). Moreover, high population density increases food consumption, respiratory rate, fat content (Applebaum and Heifetz 1999), immune function (Niemelä *et al.* 2012), the expression of heat shock proteins (HSPs), and thermal performance (Sørensen and Loeschcke 2001). In contrast, the high density could decelerate insect population growth (Meng and Li 2000, Zhang *et al.* 2019), and the morphological and physiological characteristics of *Agrotis ipsilon* have been shown to remain unchanged at high density (Sappington and Showers 1992).

These different views indicate that the effect of population density on insects is complex, and further experimental evidence is needed. In addition, most studies on the population density of insects have been more concerned with constant temperature and have paid less attention to the role of temperature variability.

*Sitobion avenae* (Fabricius) is one of the most destructive wheat pests (George and Gair 1979, Watt *et al.* 1984, Larsson 2005, Zhao *et al.* 2019) and is widely distributed in tropical and cool temperate regions, where temperature variability and different population densities are common (Winder *et al.* 1999). In this species, constant temperature, alternating temperature, and different mean temperatures with the same temperature amplitude are known to affect life history traits, such as growth, development, survival, and reproduction (Sigsgaard 2000, Zhao *et al.* 2017, Cao *et al.* 2018, Zhao *et al.* 2019). However, the interactive effects of temperature amplitude and population density have rarely been examined. Considering this scenario, the present study addressed two questions: whether population density and temperature amplitude have an interactive effect; and how population density and temperature amplitude affect development, survival, longevity, and fecundity of *S. avenae*.

## Materials and Methods

### Insect Rearing

Samples of the aphid *S. avenae* were collected from a wheat test field (116°16′ N, 35°55′ E) in Linfen, Shanxi Province, in May 2016. No insecticide had been applied to the wheat field during the preceding years. Aphids were reared on 10- to 20-cm tall winter wheat seedlings in screen cages (60 × 60 × 60 cm) at 22 ± 5°C with 50–60% relative humidity and an artificial photoperiod of 16: 8 (L:D) h. Before the experiment, *S. avenae* aphids had been bred under these conditions for at least 3 y.

### Design and Manipulation of Temperature Amplitude Treatments

**Fig. 1.**
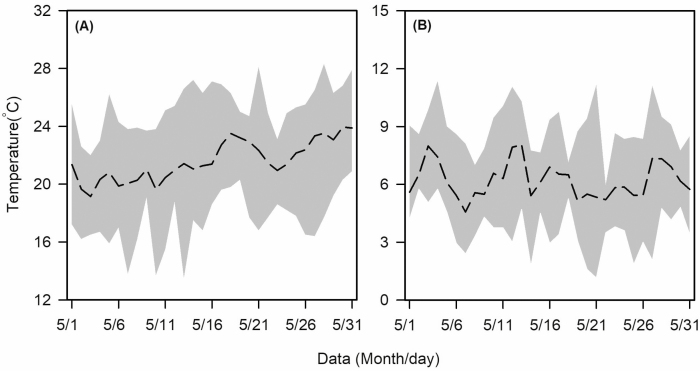
Wheat fields with diurnal mean temperatures (A) and temperature amplitudes (B) in May in 2012–2018. Dash line represents mean temperature, and gray part represents temperature range.

**Fig. 2.**
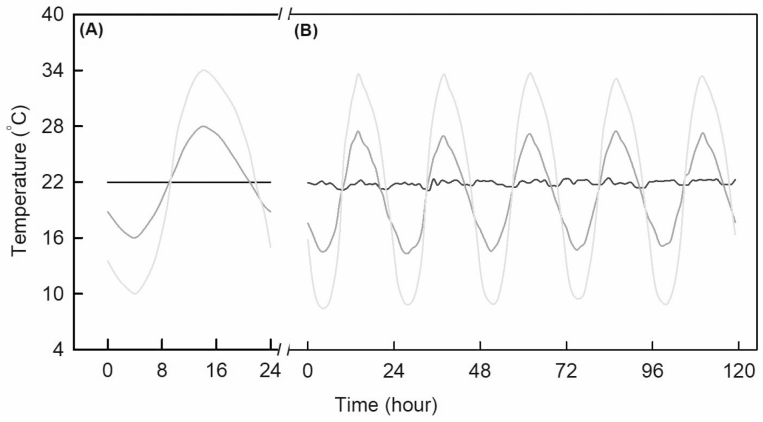
Target (A) and recorded temperatures with different temperature amplitudes in different climate chambers for five consecutive days (B).

A 24-h temperature cycle was used to simulate the mean temperatures and temperature amplitudes in a wheat field in Linfen in May from 2012 to 2018 (Fig. 1). Daytime temperature lasted for 16 h (06:00–20:00) with the daily maximum temperature (DTmax) occurring at 14:00, and the night temperature lasted for 8 h (20:00–06:00) with the daily minimum temperature (DTmin) occurring at 4:00. Temperature changed gradually between DTmax and DTmin. Three temperature amplitudes were used: 22 ± 0, 22 ± 6, and 22 ± 12°C (Fig. 2A). We selected 22 ± 12°C as the widest diurnal range because summer temperatures were mostly within this range in the microhabitat that this species experiences in wheat fields in Beijing. The daily mean temperature experienced in these fields was approximately 22°C. Temperatures in the climate chambers were logged every 20 min using an Onset HOBO Pro v2 data logger (U23-001, Bourne, MA; Table 1, Fig. 2B). For the experiment, the average temperature in each chamber was approximately 22°C, with a relative humidity of 30–50%. The photoperiod of 16: 8 (L:D) was maintained.

**Table 1.** Target and actual recorded temperatures with different temperature amplitudes around 22°C in climate chambers

Target temperature range (± °C)	Recorded temperature (mean ± SD)	
	Average (°C)	Temperature amplitudes (°C)
0	22.39 ± 0.13	0.89 ± 0.20
6	21.21 ± 0.26	6.89 ± 0.15
12	21.85 ± 0.20	11.93 ± 0.28

### Experimental Protocol

At the beginning of the experiment, the newly born, uniformly sized nymphs (less than 6-h old) were placed into clip cages (2.5-cm diameter × 2.0-cm height) and then fixed on five to six leaves of wheat seedlings at densities of 1, 5, and 10 nymphs (D1, D5, and D10, respectively). The host plants with the experimental aphids were moved into different chambers. We used three replicates per temperature treatment for each density group, with ten cages per replicate of D1, five cages per replicate of D5, and four cages per replicate of D10. The status of the aphids in each cage was recorded twice a day, at 08:00 and 20:00, because the temperatures in the climate chambers at these times were similar to the room temperature (approximately 22°C). When the aphids had developed to the adult stage, the number of offspring and survival of adults in each cage were checked daily at 20:00. Mortality in the clip cages was kept low in the absence of stress by 1) using well-ventilated clip cages to keep the aphids from escape; 2) cleaning out exuviates and newly born nymphs without disturbing the tested aphids during daily checking; and 3) transferring the tested aphids carefully to fresh seedlings every 2–3 d. The experiment lasted 1 mo.

### Statistical Analyses

All statistical analyses were performed using SPSS (SPSS 19.0; Chicago, IL). Means were compared using Tukey’s HSD test. Differences among different temperature amplitudes or densities were considered to be significant at *P* < 0.05. Normal probability plots revealed that the data were approximately normally distributed; hence, no transformations were performed. Effects of temperature amplitude and population density on development, survival, longevity, and fecundity were analyzed using multiple analysis of variance, in which temperature amplitude and population density were treated as fixed factors. ‘Early-instar’ and ‘late-instar’ nymphs refer to 1–2 and 3–4 instar nymphs, respectively. The development duration of nymphs was estimated for individuals that were alive when entering the next stage. Survival rate was estimated as the percentage of individuals alive when entering the next stage. Fecundity was defined as the number of nymphs per adult female. Longevity was defined as the number of days the adults survived.

## Results

**Fig. 3.**
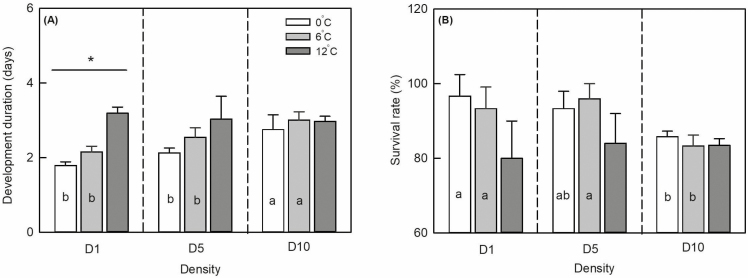
Mean development duration (A) and survival rate (B) of *Sitobion avenae* early-instar nymphs at different population densities. White, gray, and deep gray columns represent temperature amplitudes of ±0, ±6, and ±12°C, respectively. Vertical bars indicate ±SD. ‘*’ represents significant differences among different temperature amplitudes within each density at *P* < 0.05 level. Different lowercase letters in the columns indicate significant differences among treatments at *P* < 0.05 level.

Population density (*F*_2,27_ = 8.365, *P* = 0.003) and temperature amplitude (*F*_2,27_ = 20.343, *P* < 0.001) as well as their interaction (*F*_4,27_ = 4.103, *P* = 0.016) significantly influenced the development duration of early-instar nymphs (Table 2, Fig. 3). At different population densities, the development duration of early-instar nymphs increased gradually with the increasing temperature amplitude. In D1, the temperature amplitude had a significant influence on this duration (*F*_2,9_ = 79.906, *P* < 0.001) but not in D5 and D10 (*F*_2,9_ = 4.086, *P* = 0.076; *F*_2,9_ = 0.821, *P* = 0.484, respectively). At different temperature amplitudes, the development duration of early-instar nymphs increased gradually with increasing population density. At temperature amplitudes of ±0 and ±6°C, population density more strongly influenced the development duration of early-instar nymphs (*F*_2,9_ = 12.291, *P* = 0.008; *F*_2,9_ = 12.361, *P* = 0.007) but the difference was not significant at ±12°C (*F*_2,9_ = 0.280, *P* = 0.765).

**Table 2.** Results of the analyses of variance for effects of temperature amplitudes and population densities on the development duration, survival rate, longevity, and fecundity of *Sitobion avenae*

Trait	Source	df	MS	*F*	*P*
Early-instar nymph development duration	Population Density (PD)	2,27	0.661	8.365	0.003
	Temperature amplitudes (TA)	2,27	1.609	20.343	<0.001
	PD × TA	4,27	0.324	4.103	0.016
Early-instar nymph survival rate	Population Density (PD)	2,27	125.231	3.959	0.038
	Temperature amplitudes (TA)	2,27	220.954	6.986	0.006
	PD × TA	4,27	66.787	2.112	0.121
Later-instar nymph development duration	Population Density (PD)	2,27	0.360	4.049	0.035
	Temperature amplitudes (TA)	2,27	1.037	11.661	0.001
	PD × TA	4,27	0.028	0.313	0.865
Later-instar nymph survival rate	Population Density (PD)	2,27	62.787	1.594	0.230
	Temperature amplitudes (TA)	2,27	187.120	4.752	0. 022
	PD × TA	4,27	84.620	2.149	0.116
Longevity	Population Density (PD)	2,27	18.459	28.794	<0.001
	Temperature amplitudes (TA)	2,27	1.412	2.202	0.139
	PD × TA	4,27	2.533	3.952	0.018
Fecundity	Population Density (PD)	2,27	8.081	1.395	0.273
	Temperature amplitudes (TA)	2,27	95.276	16.450	<0.001
	PD × TA	4,27	20.583	3.554	0.026

Population density (*F*_2,27_ = 3.959, *P* = 0.038) and temperature amplitude (F_2,27_ = 6.986, *P* = 0.006) significantly affected early-instar nymph survival, whereas their interaction did not (*F*_4,27_ = 2.112, *P* = 0.121; Table 2, Fig. 3). At different population densities, there were no significant differences in early-instar nymph survival with different temperature amplitudes (±0, ±6, and ±12°C; *F*_2,9_ = 4.200, *P* = 0.072; *F*_2,9_ = 3.526, *P* = 0.097; *F*_2,9_ = 0.375, *P* = 0.702). At ±0 and ±6°C, early-instar nymph survival significantly decreased with increasing population density (*F*_2,9_ = 5.945, *P* = 0.038; *F*_2,9_ = 6.960, *P* = 0.027). At a temperature amplitude of ± 12°C, the population density had no significant influence on their survival rate (*F*_2,9_ = 0.291, *P* = 0.758). Early-instar nymph survival rate was 4.0 and 4.2% higher at temperature amplitudes ±6 and ±12°C, respectively, than that at 0°C.

**Fig. 4.**
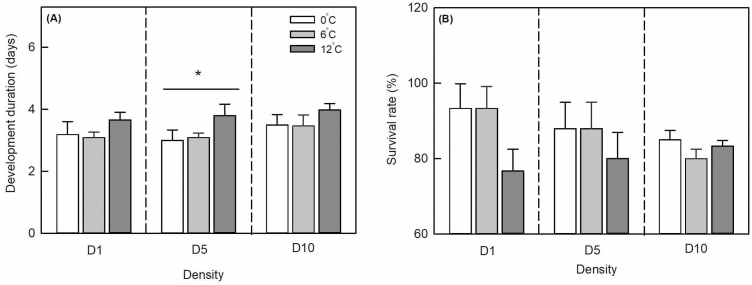
Mean development duration (A) and survival rate (B) of *Sitobion avenae* late-instar nymphs at different population densities. White, gray, and deep gray columns represent temperature amplitudes of ± 0, ±6, and ±12°C, respectively. Vertical bars indicate ±SD. ‘*’ represents significant differences among different temperature amplitudes within each density at *P* < 0.05 level.

Population density (*F*_2,27_ = 4.049, *P* = 0.035) and temperature amplitude (*F*_2,27_ = 11.661, *P* < 0.001) significantly affected the development duration of late-instar nymphs, but their interaction did not (*F*_4,27_ = 0.313, *P* = 0.865; Table 2, Fig. 4). At different population densities, this development duration increased with increasing temperature amplitude. In D5, temperature amplitude had a significant influence on the development duration of late-instar nymphs (*F*_2,9_ = 6.461, *P* = 0.032), but this influence was not significant in D1 and D10 (*F*_2,9_ = 3.132, *P* = 0.117 and *F*_2,9_ = 2.643, *P* = 0.150, respectively). At different temperature amplitudes (±0, ±6, and ±12°C), population density did not significantly affect the development duration of late-instar nymphs (*F*_2,9_ = 1.474, *P* = 0.301; *F*_2,9_ = 2.559, *P* = 0.157; *F*_2,9_ = 1.001, *P* = 0.442).

Temperature amplitude (*F*_2,27_ = 4.752, *P* = 0. 022) significantly affected late-instar nymph survival but not population density (*F*_2,27_ = 1.594, *P* = 0.230) or their interaction (*F*_4,27_ = 2.149, *P* = 0.116; Table 2, Fig. 4). In D1, D5, and D10, the survival rate of late-instar nymphs decreased with increasing temperature amplitude but without significance (*F*_2,9_ = 4.167, *P* = 0.073; *F*_2,9_ = 4.200, *P* = 0.072; and *F*_2,9_ = 1.333, *P* = 0.332, respectively). At temperature amplitudes ±0 and ±6°C, the survival rate of late-instar nymphs in D1 and D5 was higher than in D10 but without significance (*F*_2,9_ = 1.040, *P* = 0.409 and *F*_2,9_ = 4.628, *P* = 0.091, respectively). The survival rate of late-instar nymphs increased with increasing population density at ± 12°C but without significance (*F*_2,9_ = 1.199, *P* = 0.365).

**Fig. 5.**
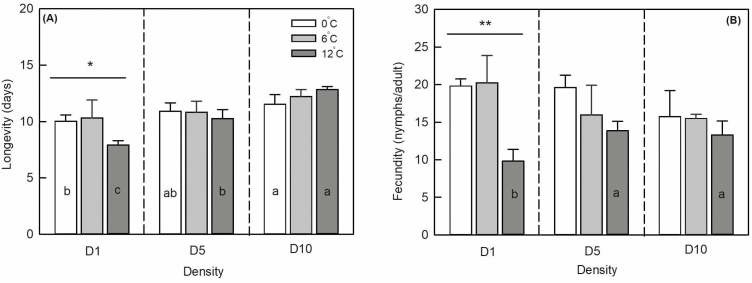
Mean longevity (A) and fecundity (B) of *Sitobion avenae* at different population densities. White, gray, and deep gray columns represent temperature amplitudes of ±0, ±6, and ±12°C, respectively. Vertical bars indicate ± SD. ‘*’ represents significant differences among the different temperature amplitudes within each density at *P* < 0.05 level. Different lowercase letters in the columns indicate significant differences among treatments at *P* < 0.05 level.

Population density (*F*_2,27_ = 28.794, *P* < 0.001) and the interaction between density and temperature amplitude (*F*_4,27_ = 3.952, *P* = 0.018) significantly affected longevity, whereas temperature amplitude alone did not (*F*_2,27_ = 2.202, *P* = 0.139; Table 2, Fig. 5). In D5 and D10, the temperature amplitude had no significant influence on longevity (*F*_2,9_ = 0.599, *P* = 0.579; *F*_2,9_ = 3.253, *P* = 0.110). However, in D1, longevity under temperature amplitudes ±0 and ±6°C was significantly higher than that under ±12°C (*F*_2,9_ = 5.520, *P* = 0.044) by 1.89 and 2.42 d, respectively. Longevity increased with increasing population density at different temperature amplitudes. At ±0 and ±12°C, population density had a significant influence on longevity (*F*_2,9_ = 5.982, *P* = 0.037 and *F*_2,9_ = 66.652, *P* < 0.001, respectively); however, population density did not affect longevity at ± 6°C (*F*_2,9_ = 2.319, *P* = 0.179).

Population density (*F*_2,27_ = 1.395, *P* = 0.273) had no significant effect on fecundity, whereas temperature amplitude (*F*_2,27_ = 16.450, *P* < 0.001) and their interaction (*F*_4,27_ = 3.554, *P* = 0.026) did (Table 2, Fig. 5). In D1, the fecundity under temperature amplitudes ±0 and ±6°C was significantly higher than that under ±12°C (*F*_2,9_ = 16.450, *P* < 0.001) by 10.00 and 10.45 nymphs/adult, respectively. However, in D5 and D10, temperature amplitudes ±0, ±6, and ±12°C did not significantly affect the fecundity (*F*_2,9_ = 2.304, *P* = 0.181 and *F*_2,9_ = 1.124, *P* = 0.385, respectively). At ±0 and ±6°C, population density had no significant effect on fecundity (*F*_2,9_ = 2.704, *P* = 0.146 and *F*_2,9_ = 2.149, *P* = 0.198, respectively). At temperature amplitude ±12°C, the fecundity in D5 and D10 was significantly higher than that in D1 (*F*_2,9_ = 5.728, *P* = 0.041) by 4.03 and 3.48 nymphs/adult, respectively.

## Discussion

### Effects of Population Density Under Constant Temperature

At constant temperature, population density significantly affected the development and survival of early-instar nymphs; the development duration was significantly higher by 0.34 and 0.97 d, respectively, in D5 and D10 than that in D1. Moreover, in D1, D5, and D10, survival was 96.67, 93.33, and 85.00%, respectively. Thus, early-instar nymph growth and survival were inhibited by increasing population density. This finding is essentially consistent with that in the literature: insect population density significantly affects their development (Woodhead and Paulson 1983, Hooper *et al.* 2003, Ma *et al.* 2010) and life history traits such as survival (Kong *et al.* 2013, Silva *et al.* 2016). This phenomenon may be attributed to intensified food competition, reduced living space, and increased quantities of excreta when population density is too high, which reduces habitat quality and causes increased development duration and reduced individual survival (Stiling 1988).

At constant temperature, high population density significantly increased longevity. There are currently three views on the correlation between population density and longevity. First, as population density increases, adult longevity increases; this has been demonstrated in, for example, *Spodoptera exigua* female adults (Wang *et al.* 2008), *Plutella xylostella* female adults (Kong *et al.* 2013), and *P. operculella* (Ma *et al.* 2010). Furthermore, adult longevity gradually decreases with increasing population density in, for example, *Aphis gossypii*, *Acyrthosiphon gossypii* (Gao *et al.* 2012), and *D. virgifera* (Branson *et al.* 1985). Additionally, longevity is the highest in adults at a certain middle population density in, for example, *Athetis lepigone* (Yan *et al.* 2014), *P. xylostella* males (Kong *et al.* 2013), *S. exigua* males (Wang *et al.* 2008), and *Loxostege sticticalis* (Kong *et al.* 2011). In our study, longevity was 1.14 and 1.76 d higher, respectively, in D5 and D10, than that in D1. This may be closely related to adult reproductive capacity. Under high-density conditions, individuals may consume more of their nutrient reserves to cope with deteriorating conditions, which consequently reduces their reproduction, thereby prolonging adult longevity (Sisodia and Singh 2002, Cingolani et al. 2020). In our study, aphid fecundity decreased gradually with increasing population density.

### Effects of Temperature Amplitude Under Low Density

Temperature amplitude significantly influenced early-instar nymph development at low population density. In D1, early-instar nymph development duration was 0.37 and 1.40 d higher at temperature amplitudes of ±6 and ±12°C, respectively, than at constant temperature. This indicates that wide temperature amplitude inhibited early-instar nymph development. This may be owing to the linear progression of insect development within the appropriate temperature amplitudes, with the growth increment at daytime temperatures being equal to that at night temperatures (Ruel and Ayres 1999, Xing *et al.* 2014, Zhao *et al.* 2014). At the widest temperature amplitude (±12°C), the highest temperature was 34°C for 6 h, which is higher than the upper temperature limit for wheat aphid development (30°C) (Dean 1974, Acreman *et al.* 1989, Lykouressis *et al.* 2009), and early-instar development may have been inhibited. However, it is worth noting that insect development continues slowly at temperatures above this upper limit, rather than stopping completely (Xing et al. 2014, 2019). However, temperature amplitude did not significantly affect late-instar nymph development. This may be because late-instar nymphs have better heat-resistance capacity than the early-instar nymphs (Zhao *et al.* 2019).

Temperature amplitude had little effect on nymph survival at low density. At constant temperatures (20–22°C), nymph survival on leaves and ears does not change significantly (at 88.9–95.5%); however, at 30°C, it decreases to 22.2–20.5%, (Acreman *et al.* 1989). Previous work indicates that nymph survival is zero at a constant temperature of 30°C (Dean 1974). However, even at the highest temperature amplitude (±12°C, with daily maximum temperatures of 34°C), we observed survival rates of 80.0 and 76.7% for early- and late-instar nymphs, respectively, far exceeding the reported 30°C threshold. This difference may be because, at night temperatures, the adult metabolic rate slows, and the production of protective substances, such as HSP, mannitol, and sorbitol, increases (Zerebecki and Sorte 2011), which may, to a certain extent, repair thermal damage in adults (Yang and Stamp 1995, Xing *et al.* 2015a). Further, we found that adult survival and reproduction occurred even at the widest temperature amplitude, in contrast to the findings of Dean (1974). Similarly, this may be a result of recovery from daytime thermal damage at cooler night temperatures (Zhao *et al.* 2014).

### Interactive Effects of Population Density and Temperature Amplitude

As population density increased, the negative effects under the widest amplitude gradually declined and even reversed. At the lowest density, early-instar nymph development duration at ±12°C temperature amplitude was 1.40 d greater than that at constant temperature; at the highest density, in contrast, it was only 0.22 d greater than that at constant temperature. This shows that an increasing population density counteracts the inhibitory effects of wide temperature amplitude on the development of early-instar nymphs. At the lowest population density, longevity was 1.89 d lower at ± 12°C temperature amplitude than that at constant temperature; however, at the highest population density, longevity was 1.28 d greater at ±12°C than that at constant temperature. This shows that high population density positively affects longevity under wide temperature amplitude; we observed similar findings for fecundity and nymph survival. These findings are in contrast to those of prior studies on the effects of temperature amplitude and population density on insects. Wide temperature amplitude has been shown to inhibit development (Xing *et al.* 2014, Verheyen *et al.* 2019) and reduce survival (Xing *et al.* 2019), longevity (Cao *et al.* 2018), and fecundity (Carrington *et al.* 2013, Stoks *et al.* 2017); high population density has produced similar results (Hooper *et al.* 2003, Gao *et al.* 2012, Kong *et al.* 2013, Silva *et al.* 2016). However, we found that with increasing population density, differences in nymph development and survival between the constant temperature and wide temperature amplitude treatments gradually decreased. This may be attributed to the increased energy demands associated with development, causing increased competition for food and space among nymphs and resulting in more stress at a high population density (Sørensen and Loeschcke 2001, Wang *et al.* 2007, Henry *et al.* 2018). Nonetheless, the upregulation of HSP expression and other protective substances at night can reduce the adverse effects of high temperature in wide amplitudes and the adverse effects of high population density. This shows that a response to one environmental pressure may have a synergistic effect on responses to other pressures (Liess *et al.* 2016). Such pressures may include external pressures such as high or low ambient temperature, pesticide exposure (Delnat *et al.* 2019), and intraspecific pressures such as population density.

### Pest Control Application

Our results show that compared to constant temperature, wide daily temperature amplitudes greatly influence insect development, survival, longevity, and fecundity. Further, the combined effects of temperature amplitude and population density are complex and do not necessarily have a negative impact on insect life history. In previous studies, the traditional linear model of ‘constant temperature development’ was always used to assess pest population dynamics (Wiman *et al.* 2014, Walter *et al.* 2018); however, these modes cannot describe pest population dynamics at wide temperature ranges. Additionally, high density and wide temperature amplitude always negatively affect pest population dynamics, respectively. However, in nature, both influences exist simultaneously. To improve prediction accuracy for pest population dynamics and damage assessment, we suggest that temperature amplitude and population density be included in pest development or survival models.
